# Effect of Inpatient Multicomponent Occupational Rehabilitation Versus Less Comprehensive Outpatient Rehabilitation on Sickness Absence in Persons with Musculoskeletal- or Mental Health Disorders: A Randomized Clinical Trial

**DOI:** 10.1007/s10926-017-9708-z

**Published:** 2017-04-11

**Authors:** Lene Aasdahl, Kristine Pape, Ottar Vasseljen, Roar Johnsen, Sigmund Gismervik, Vidar Halsteinli, Nils Fleten, Claus Vinther Nielsen, Marius Steiro Fimland

**Affiliations:** 1 Department of Public Health and Nursing, Faculty of Medicine and Health Sciences, Norwegian University of Science and Technology, NTNU, Trondheim, Norway; 2 Department of Physical Medicine and Rehabilitation, St. Olavs Hospital, Trondheim University Hospital, Trondheim, Norway; 3 Regional Center for health care improvement, St. Olavs Hospital, Trondheim University Hospital, Trondheim, Norway; 4 Department of Community Medicine, UiT The Artic University of Norway, Tromsø, Norway; 5 Department of Public Health, Aarhus University, Aarhus, Denmark; 6 Hysnes Rehabilitation Center, St. Olavs Hospital, Trondheim University Hospital, Trondheim, Norway

**Keywords:** Return to work, Sick leave, Musculoskeletal diseases, Mental health, Cognitive therapy

## Abstract

*Purpose* To assess effects of an inpatient multicomponent occupational rehabilitation program compared to less comprehensive outpatient rehabilitation on sickness absence in persons with musculoskeletal- or mental health disorders.* Methods* Randomized clinical trial with parallel groups. Participants were individuals 18–60 years old on sick-leave for 2–12 months with a sick-leave diagnosis within the musculoskeletal, psychological or general and unspecified chapters of ICPC-2, identified in a national register. The inpatient program (4 + 4 days) consisted of Acceptance and Commitment Therapy (ACT), physical training and work-related problem-solving including creating a return to work plan and a workplace visit if considered relevant. The outpatient program consisted primarily of ACT (6 sessions during 6 weeks). Both programs were group based. Primary outcome was cumulated number of sickness absence days at 6 and 12 months follow-up. Secondary outcome was time until sustainable return to work.* Results* 168 individuals were randomized to the inpatient program (n = 92) or the outpatient program (n = 76). We found no statistically significant difference between the programs in median number of sickness absence days at 6 and 12 months follow-up. In the outpatient program 57% of the participants achieved sustainable return to work (median time 7 months), in the inpatient program 49% (log rank, p = 0.167). The hazard ratio for sustainable return to work was 0.74 (95% CI 0.48–1.32, p = 0.165), in favor of the outpatient program.* Conclusions *This study provided no support that the more comprehensive 4 + 4 days inpatient multicomponent occupational rehabilitation program reduced sickness absence compared to the outpatient rehabilitation program.

## Introduction

Too many people leave the workforce prematurely due to health problems or disability, and too few workers with health problems are able to stay in work [1], particularly due to musculoskeletal and mental health disorders [2]. In addition to individual suffering this causes considerable costs for society. In Norway 5% of the gross domestic product is spent on disability and sickness benefits [1].

Several treatment and rehabilitation programs to facilitate work participation have been investigated, notably for persons with low back pain, but also for common mental disorders [3–6]. However, previous studies have been performed in outpatient settings, whereas there is a long tradition for inpatient multicomponent occupational rehabilitation in Norway. These programs usually consist of cognitive behavioral therapy, physical exercise and patient education [7], but little workplace involvement [8]—which is considered important in improving return to work rates [9–11]. There is some support for including these components in such programs. Cognitive behavioral therapy is recommended for patients with chronic low back pain [12] and common mental health disorders [13], and is often included in return to work interventions [14]. Physical exercise provides substantial health benefits [15, 16] and is also inversely associated with disability pension [17] and sickness absence [18]. Patient education is considered beneficial in treatments of chronic low back pain [12] and common mental health disorders [19], and often included in return to work programs [14, 20]. Still, no randomized studies have assessed the effect of inpatient multicomponent occupational rehabilitation on work participation.

The diagnosis-specific emphasis of previous studies [3–5, 9, 21], is somewhat in contrast to the increasing documentation of overlap between musculoskeletal complaints and mental health problems [22, 23], and the fact that return to work rehabilitation programs for low back pain also have been suggested to be useful for persons on sick leave with mental health disorders [24]. In line with this, occupational rehabilitation centers in Norway include different diagnostic groups in the same program [7]. However, we are not aware of studies evaluating return to work rehabilitation programs for both somatic and mental health disorders with a rigorous study design.

Although inpatient occupational rehabilitation programs in Norway typically last about 4 weeks where patients live at the centers, there are several reasons for investigating different approaches: (1) in a pilot-investigation, several participants reported that 4 weeks was too long to stay away from home; (2) a continuous stay at a rehabilitation center does not allow for workplace involvement, and (3) a 4-week rehabilitation period is based on traditions rather than scientific evidence, and less costly alternatives should be investigated. Hence, we designed a randomized study investigating effects on sick leave of an inpatient multicomponent occupational rehabilitation program lasting 4 + 4 days, separated by 2 weeks where a workplace visit could be performed. The comparative program was a less comprehensive outpatient program, consisting mainly of a recent form of cognitive behavior therapy [25]. We hypothesized that the inpatient multicomponent occupational program would reduce sickness absence more than the less comprehensive outpatient program, as it in addition to cognitive behavioral therapy, included physical training, patient education, a return to work plan and a workplace visit when relevant.

## Methods

### Study Design and Participants

We conducted a randomized clinical trial with parallel groups, comparing an inpatient multicomponent occupational program with a single-component outpatient program (hereafter also referred to as the inpatient- and outpatient program, respectively) for individuals on sick-leave due to musculoskeletal-, unspecific-, or common mental health disorders. Details about the study design have been published in a protocol article [8]. The study was approved by the Regional Committee for Medical and Health Research Ethics in Central Norway (No.: 2012/1241), and the trial is registered in https://clinicaltrials.gov/ (No.: NCT01926574). The results are presented according to the CONSORT statement [26].

Eligible participants were 18 to 60 years of age sick listed 2 to 12 months with a diagnosis within the musculoskeletal (L), psychological (P) or general and unspecified (A) chapters of the ICPC-2 (International Classification of Primary Care, Second edition). The current sick leave status had to be at least 50% off work. Exclusion criteria, assessed by a comprehensive questionnaire and an outpatient screening performed by a physician, physiotherapist and psychologist, were: (1) alcohol or drug abuse; (2) serious somatic (e.g. cancer, unstable heart disease) or psychological disorders (e.g. high suicidal risk, psychosis, ongoing manic episode); (3) specific disorders requiring specialized treatment; (4) pregnancy; (5) currently participating in another treatment or rehabilitation program; (6) insufficient oral or written Norwegian language skills to participate in group sessions and fill out questionnaires; (7) scheduled for surgery within the next 6 months; and (8) serious problems with functioning in a group setting.

### Interventions

*The inpatient program* consisted of several components; group-based cognitive behavioral therapy, individual and group-based physical training, mindfulness, psychoeducation on stress and individual meetings with the coordinators for work-related problem-solving and creating a return to work plan. The cognitive behavioral approach was Acceptance and commitment therapy (ACT), which is a new form of cognitive behavioral therapy that emphasizes accepting both negative and positive experiences while using the individuals` values to guide them towards their goals [25]. Studies have suggested an effect of ACT on the main causes of sickness absence, namely chronic pain [27], anxiety [28] and depression [28, 29]. Through mindfulness techniques, values and committed action the aim of ACT is to increase psychological flexibility [30] and to increase return to work by increasing coping and motivation, as indicated by a randomized pilot study [31]. ACT was chosen as the cognitive behavioral therapy-approach in this study because of its transdiagnostic approach [32].

The intervention lasted four full workdays in week 1 and week 4 (8 days in total; 6–7 h each day) during which the participants resided at the rehabilitation center, separated by 2 weeks at home (week 2 and 3). The 2 weeks at home included at least two contacts with the team coordinator (in person or by telephone) and a meeting with the employer if regarded relevant and the participant gave permission. The coordinators who mentored the participants were supervised by a certified ACT-instructor before and during (monthly) the intervention. The program took place at Hysnes rehabilitation center, established as part of St. Olavs Hospital, in central Norway.

*The outpatient program* consisted primarily of one component; group-based ACT. The sessions were held at the Department of Physical Medicine and Rehabilitation at St. Olavs Hospital once a week for 6 weeks, each session lasting 2.5 h. The sessions were led by either one of two physicians (specialists in Physical medicine and rehabilitation) or a psychologist; all supervised by the same ACT instructor as the coordinators in the inpatient program. The participants were given assignments to practise at home between sessions, including a daily 15 min audio-guided mindfulness practice. In addition the participants were offered two individual sessions with a social worker experienced in occupational rehabilitation and trained in ACT to clarify personal values and work-related issues. The program also included a motivational group discussion with a physiotherapist on the benefits of physical training. An individual session with both the social worker and group leader present ended the program. In this session a summary letter was written to the participant’s general practitioner.

### Study Context

All legal residents in Norway are included in the Norwegian public insurance system. Medically certified sick leave is compensated with 100% coverage for the first 12 months. The first 16 days are covered by the employer, the rest by the Norwegian Welfare and Labour Administration. After 12 months of sick leave it is possible to apply for the more long-term medical benefits, work assessment allowance and disability pension, which both covers approximately 66% of the income. Individuals on work assessment allowance are supposed to work according to their workability.

### Outcome Measures

Participants were followed for 12 months after inclusion. During this period, sickness absence was registered in monthly intervals, both as number of days per month and as a dichotomous measure of whether or not the participant was registered on sick leave that month. Outcomes were measured using data from the National Social Security System Registry, where all individuals receiving any form of benefits in Norway are registered by their social security number. The data consisted of registrations of medical benefits from four different sources; sick-leave payments, sick leave certificates, work assessment allowance and disability pension. Monthly intervals (rather than exact dates) were used in order to include all relevant sick leave benefits in the same measure, as exact dates were not available for payments and the long-term benefits. Work assessment allowance was adjusted for delay in payments up to 2 months.

The primary outcome measure was cumulated number of sickness absence days, calculated at 6 and 12 months after inclusion. By combining information from the different medical benefits we calculated days on medical benefits (according to a 5-day work week) for every month during follow-up. Time on graded sick leave was transformed to whole workdays. Days receiving sick-leave payment and work assessment allowance were adjusted for employment fraction, including a graded disability pension at inclusion. Any increase in disability pension during follow-up was counted as sick leave.

The secondary outcome measure was time until full sustainable return to work defined as 1 month without relapse, i.e. one monthly interval not receiving any medical benefits (except any graded disability the participant had when entering the study).

Questionnaires measuring baseline characteristics like education, pain, anxiety and depression symptoms were answered by the participants before the screening. Anxiety and depression were assessed using The Hospital Anxiety and Depression Scale (HADS) [33]. It consists of 14 items, where seven items measure anxiety and seven depression symptoms. It is scored on a 4-point Likert scale according to intensity of symptoms in the last week. The maximum score is 21 on each subscale. To assess pain we used one question from the Brief Pain Inventory [34]. The participants were asked to grade the average pain during the last week on a 0 (no pain) to 10 (worst imaginable pain) numeric rating scale.

### Randomization

**Fig. 1:**
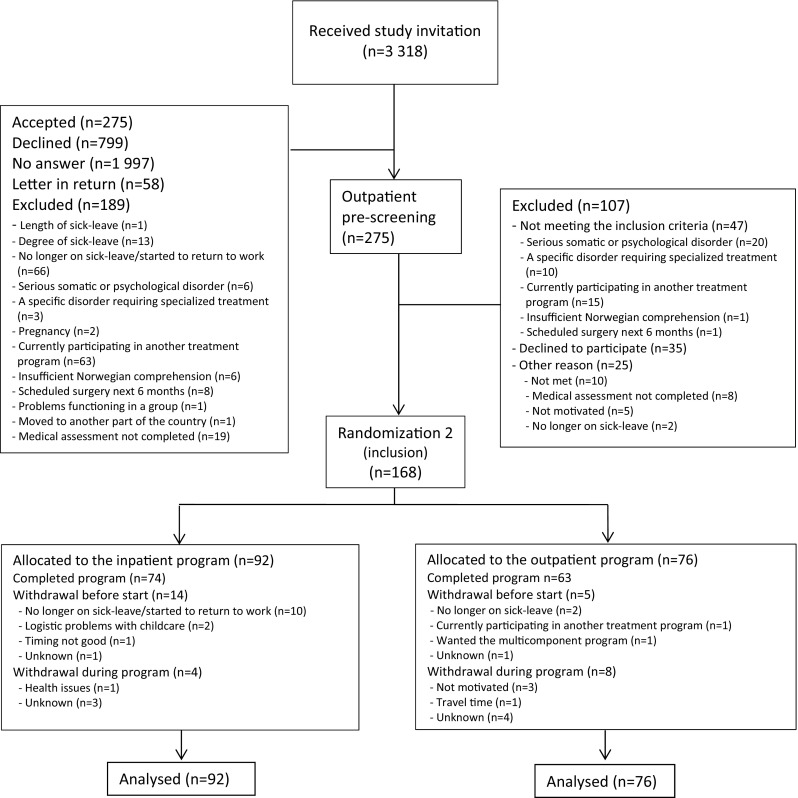
Participant flow through the study

Invited participants completed a short questionnaire assessing initial eligibility. Those eligible were invited for an outpatient screening assessment. If the screening was passed (Fig. 1), subjects were randomized to either the inpatient or the outpatient program. A flexibly weighted randomization procedure was provided by the Unit of Applied Clinical Research (third-party) at the Norwegian University of Science and Technology, to ensure that the rehabilitation center had enough participants to run monthly groups in periods of low recruitment. This affected group-sizes differentially, and therefore the researchers were not blinded. Sickness absence data was registered and provided by employees at the Norwegian Welfare and Labor Service whom were unaware of group allocation. It was not possible to blind neither the participants nor the caregivers for treatment.

### Sample Size

Sample size calculations were based on three approaches [8]:

1. Comparison of RTW with Kaplan Meier survival analysis with log rank test with a hazard ratio of 0.6 (alpha 0.05, beta 0.20) would require 63 in each group.
2. Comparison of number of days with sick leave at 6 months of follow-up (p = 0.05; 90% power): An average of 60 days (SD 40) and 90 days (SD 60) of sick leave in the intervention and comparative group, respectively would require 61 persons for each group.
3. Comparing ratios of participants at work after 1 year of follow-up with the same statistical assumptions as point 2; and a difference of 60 versus 40% RTW, would require 63 people in each group.

With an estimated 20% loss to follow-up we aimed to include 80 persons in each arm. The sample size calculations were based on results from previous studies in this field [5, 9, 10].

### Statistical Analysis

Number of days of sick leave at 6 and 12 months after inclusion for the two programs were calculated and compared using the Mann–Whitney U (Wilcoxon rank sum) test. For time until sustainable return to work Kaplan Meier curves were estimated and compared with the log rank test. We estimated hazard ratios for return to work using Cox proportional hazard model with the Efron method for ties [35]. Time was calculated as number of months and participants were censored at “full sustainable return to work” or end of follow-up. We performed analyses without adjustment and with adjustment for gender, age, level of education, main diagnosis for sick leave and length of sick leave at inclusion. The proportionality hazard assumption was checked using the Schoenfeld Residual Test [36]. All analyses were performed after the “intention to treat” principle. Additional “per protocol” analyses were done by excluding participants that withdrew after randomization (before or during the programs) and/or attended less than 60% of the sessions of the outpatient program.

In addition to the main analyses, we performed several post hoc sensitivity analyses in order to account for characteristics of the sickness absence patterns and data structure which we observed in the course of the study. First, we observed that several participants alternated between being on and off benefits. We therefore performed a repeated events analysis allowing individuals to alternate between being on and off benefits every month of follow-up using general estimating equations (GEE). Secondly, we observed single months without payment in between longer periods of payments. As the Norwegian holiday lasts 5 weeks, we performed an additional sensitivity analysis on time until sustainable return to work where we defined return to work as 2 months without benefits.

We considered p-values (two-tailed) <0.05 to be statistically significant. Precision was assessed using 95% confidence intervals. All analyses were done using STATA 13.1 (StataCorp. 2013. Stata Statistical Software: Release 13. College Station, TX: StataCorp LP).

## Results

The flow of participants through the study is illustrated in Fig. 1. Between October 2012 and November 2014, 12 007 potential participants from the regional area were identified in the National Social Security System Registry and 3 318 were randomized to receive an invitation to the short program. Of these 275 accepted the invitation. After screening 168 remained and were randomized to the inpatient program (n = 92) or the outpatient program (n = 76). The groups consisted of maximum 9 participants.

For the inpatient program, 14 people withdrew before they began the program and four quit during the program. For the outpatient program, five people withdrew before the program started and eight during the program. Those who started the outpatient program attended on average 7.9 of the 10 meetings and 59 (83%) attended at least 60% of the sessions. For the inpatient program there is no data available regarding the number of sessions participants attended, but as it was an inpatient program the participants were assumed compliant if they did not withdraw. All participants were included in the analyses. A workplace visit was performed for 13% (n = 10) of the participants who started the inpatient program.

### Participants` Characteristics

Most of the participants (65%) worked full time prior to their sick-leave, while 18% worked part time, 4% had a graded disability pension and 13% had no job. The median number of days on sick-leave the last 12 months before inclusion in the study (i.e. second randomization) was 226 calendar days (interquartile range (IQR) 189–271). A musculoskeletal diagnosis was most common (52%), followed by psychological (38%) and general and unspecific (10%) diagnoses. The mean age of participants was 45 years and the majority was women (79%). The baseline characteristics of the participants in the two programs were fairly similar (Table 1).

**Table 1:** Baseline characteristics of participants

	Inpatient program (n = 92)	Outpatient program (n = 76)
Age mean (SD)	45.0 (8.7)	45.1 (9.6)
Women n (%)	71 (77%)	62 (82%)
Higher education n (%)^a^	45 (49%)	31 (41%)
Work status n (%)		
No work	15 (16%)	7 (9%)
Full time	57 (62%)	52 (68%)
Part time	15 (16%)	16 (21%)
Graded disability pension^b^	5 (5%)	1 (1%)
Sick-leave status n (%)^c^		
Full sick-leave	41 (45%)	35 (46%)
Partial sick-leave	45 (49%)	36 (47%)
Work assessment allowance	6 (7%)	5 (7%)
HADS mean (SD)		
Anxiety (0–21)	7.8 (4.4)	7.4 (4.3)
Depression (0–21)	6.7 (4.3)	6.0 (4.1)
Pain level mean (SD)		
Average pain (0–10)	4.7 (2.3)	4.6 (2.0)
Main diagnoses for sick-leave (ICPC-2) n (%)^c^		
A—general and unspecified	9 (10%)	7 (9%)
L—musculoskeletal	48 (52%)	40 (53%)
P—psychological	35 (38%)	29 (38%)
Length of sick leave at inclusion^c,d^		
Median days (IQR)	224 (189–262)	229 (187–275)

^a^Higher (tertiary) education: college or university

^b^Individuals working part time that at inclusion also received a graded disability pension

^c^Based on data from the National Social Security System Registry

^d^Number of days on sick leave during the last 12 months prior to inclusion. Measured as calendar days, not adjusted for partial sick- leave

### Sickness Absence Days

**Fig. 2:**
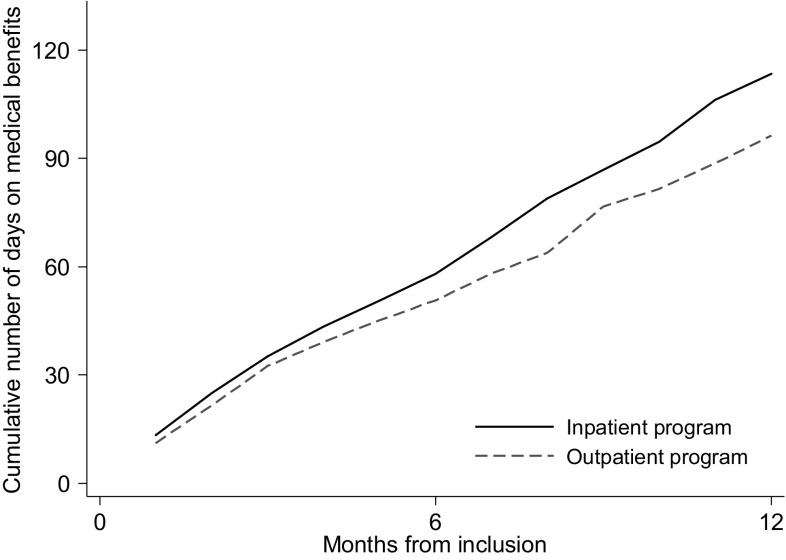
Cumulative number of days (median) on medical benefits for the inpatient- and the outpatient program during 12 months of follow-up. Adjusted for employment fraction and transformed to whole workdays according to a 5-day work week

The median number of sickness absence days (work days) at 6 months after inclusion was 58 (IQR 37–92) for the inpatient program and 51 (IQR 32–85) for the outpatient program. The difference was not statistically significant (Mann–Whitney U test, p = 0.284). For the 12 months follow-up, the median number of sickness absence days was 114 (IQR 46–172) for the inpatient program and 96 (IQR 35–175) for the outpatient program (Fig. 2). The difference was not statistically significant (Mann–Whitney U test, p = 0.403).

### Sustainable Return to Work

**Fig. 3:**
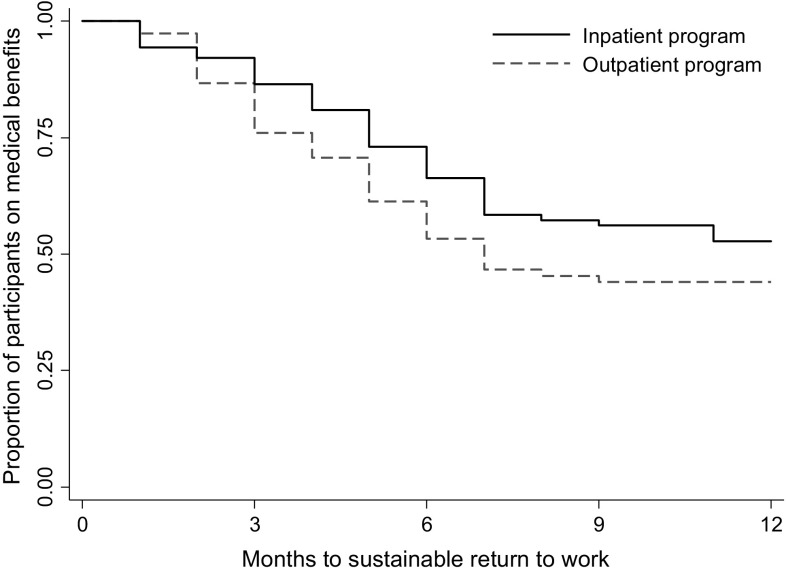
Survival curves from the Kaplan Meier analysis showing time to sustainable return to work (i.e. 1 month not receiving medical benefits) for the inpatient- and the outpatient program

In total 88 participants achieved sustainable return to work (i.e. 1 month without benefits) during 12 months follow-up, 45 participants (49%) in the inpatient program and 43 participants (57%) in the outpatient program. Median time until sustainable return to work was 7 months for the outpatient program (IQR 4-not reached). The inpatient program did not reach 50% return to work in the follow-up period (IQR 5-not reached). Figure 3 shows the Kaplan Meier plot. The difference between the programs was not statistically significant (log rank test: p = 0.167). Cox regression analysis without adjustment gave a hazard ratio of 0.74 (95% CI 0.48–1.32, p = 0.165) for sustainable return to work, in favor of the outpatient program. Adjustment for age, gender, education, main diagnosis for sick leave and length of sick leave at inclusion gave similar results (hazard ratio 0.72, 95% CI 0.46–1.11, p = 0.135).

### Other Sickness Absence Measures

Of the participants achieving sustainable return to work, 15 participants (33%) in the inpatient program and 20 (47%) participants in the outpatient program returned to medical benefits during the 12 months follow-up. At 12 months, 40 participants (43%) in the inpatient program and 30 (39%) in the outpatient program was not on medical benefits (excluding graded disability benefits). About half the participants received work assessment allowance in both groups (50 and 49% respectively) and 5% of the participants in the inpatient program and 12% in the outpatient program were on sick leave. One participant in the inpatient program received full disability pension.

**Fig. 4:**
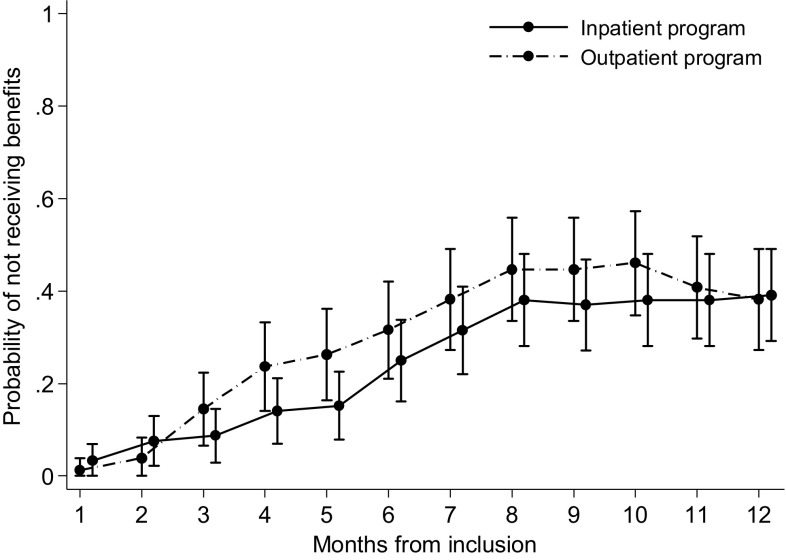
Estimated probabilities of not receiving benefits at each month during 12 months of follow-up. Results from a repeated events analysis using logistic general estimating equations (GEE)

Repeated events analyses for return to work showed no difference between the programs at any of the time points (months of follow-up) (Fig. 4). The average odds ratio over time was 0.78 (95% CI 0.49–1.24, p = 0.299) for return to work (i.e. 1 month without benefits) in favor of the outpatient program. Adjusting for aforementioned variables did not change the conclusion.

When the analyses were performed using 2 months without medical benefits as event, the sustainable return to work rate dropped slightly to 45 and 53% for the inpatient and outpatient program respectively. Unadjusted and adjusted cox regression gave hazard ratios similar to the analyses performed with1 month without benefits as the event.

Per protocol analyses comparing number of sickness absence days in the inpatient and outpatient programs showed similar results as the main analyses at 6 months: 60 (IQR 39–96) versus 53 (IQR 32–82; p = 0.187) days and at 12 months: 118 (IQR 48–181) versus 98 (IQR 39–157; p = 0.313) days. The per protocol cox regression analyses also showed similar results as the main analyses: unadjusted HR 0.71 (95% CI 0.44–1.16, p = 0.174), and adjusted HR 0.70 (95% CI 0.43–1.15, p = 0.161).

## Discussion

Among persons on sick leave with a musculoskeletal, psychological or unspecific diagnosis, this randomized trial showed no significant difference in number of sickness absence days and time to sustainable return to work following an inpatient multicomponent occupational rehabilitation program compared to a less comprehensive outpatient program.

Even though there were no statistical differences between the programs, there were some indications that participants in the outpatient program had less sickness absence days and shorter time to sustainable return to work. However, this group also had a higher fraction of recurring sickness episodes. Hence, we performed a post hoc analysis to assess the probability of receiving/not receiving monthly medical benefits throughout the 1-year follow-up period. Assessing sickness absence in this way made the between-group differences smaller, strengthening the finding of no difference between the programs.

Return to work rates in this study were lower than in some previous return to work studies [3, 5, 6]. However, those studies only included participants with musculoskeletal complaints while this study also included common mental health disorders and unspecific complaints. The participants in this study also had longer current sickness episodes than some of the previous studies [3, 6], which might indicate more complex problems. They were also invited directly through the National Social Security System and not referred by a physician. Nevertheless, the low return to work rate for the inpatient program could indicate that the program did not match their needs.

Studies have suggested that involving the workplace in return to work programs is effective for reducing sick leave for individuals on sick leave with low back pain [5, 9] and common mental health disorders [4]. The inpatient program in this study involved one workplace visit, but only when considered relevant by the participant and the rehabilitation team, and was only performed for 13% of the participants. The reasons for not performing the workplace visit were poorly registered. Focus group interviews with individuals participating in a similar but more long-lasting program at the same rehabilitation center found that few had made concrete plans for return to work at the end of the program [37]. That so few workplace visits were performed could possibly in part explain why there was no additional effect of the inpatient program compared to the outpatient program.

In a Norwegian context this was a relatively short inpatient occupational rehabilitation program (4 + 4 days), as traditional inpatient programs typically last about 4 weeks [7]. In that regard, lack of difference between the two programs could be due to the short length of the inpatient program. As the participants included in the study had median sick leave duration of more than 200 days in the year before inclusion, they might have needed a longer rehabilitation program to facilitate the return to work process. Similarly, we cannot exclude the possibility that the outpatient program potentially might have been more effective, had it been more comprehensive. However, we are not aware of studies showing added effect of more intensive programs [38, 39].

The main strength of this randomized study was the use of registry data on medical benefits, ensuring that there were no biased assessments of end-points and no missing data. Furthermore, all participants were invited from the National Social Security System, meaning there was no referral bias. However, there was a self-selection bias as to which individuals accepted the invitation to participate in the study. Accepting the invitation meant they had to be prepared to be away from family and friends during the program if allocated to the inpatient program. From more than 3000 invitations sent, only 275 individuals accepted the invitation, which limits the generalizability of the results. Even though an inclusion criterion was sick leave for at least 8 weeks, the mean length of sick leave at inclusion was more than 220 days for both programs. It could be that individuals with greater obstacles for return to work to a larger extent accepted the invitation. This assumption is strengthened by the fact that around 50% of the participants received work assessment allowance at 12 months follow-up. As this medical benefit provided after 1 year of sickness absence only reimburses 66% of the salary compared to 100% for sick leave pay, there is a considerable financial incentive for returning to work within 1 year. As there is no randomized usual care control group we do not know if the programs reduced sick leave and increased return to work compared to usual care. Another limitation was that the researchers were not blinded. However, sickness absence was registered and provided by employees at the Norwegian Welfare and Labor Service whom were unaware of group allocation.

## Conclusion

Among persons on sick leave with a musculoskeletal, psychological or unspecific diagnosis, this study provides no support that the 4 + 4 days inpatient multicomponent occupational rehabilitation program reduces sickness absence compared to a less comprehensive outpatient program. As the inpatient program was more resource-demanding we do not recommend it to be implemented in regular clinical practice. Considering that this program was relatively short in an inpatient setting, future studies should investigate effects of more extensive inpatient occupational rehabilitation programs.
